# Mitochondrial priming in therapy-induced senescence: implications for CAR-T/NK immunosenolytic therapy

**DOI:** 10.3389/fimmu.2025.1695244

**Published:** 2025-11-07

**Authors:** Javier A. Menendez, Ruth Lupu, Begoña Martin-Castillo, Josep Sardanyés, Tomás Alarcón, Sara Verdura, Elisabet Cuyàs

**Affiliations:** 1 Program Against Cancer Therapeutic Resistance (ProCURE), Catalan Institute of Oncology, Girona, Spain; 2 Metabolism and Cancer Group, Girona Biomedical Research Institute (IDIBGI), Girona, Spain; 3 Division of Experimental Pathology, Department of Laboratory Medicine and Pathology, Mayo Clinic, Rochester, MN, United States; 4 Mayo Clinic Cancer Center, Rochester, MN, United States; 5 Department of Biochemistry and Molecular Biology Laboratory, Rochester, MN, United States; 6 Mayo Clinic Laboratory, Rochester, MN, United States; 7 Unit of Clinical Research, Catalan Institute of Oncology, Girona, Spain; 8 Centre de Recerca Matemàtica (CRM), Barcelona, Spain; 9 Institució Catalana de Recerca i Estudis Avançats (ICREA), Barcelona, Spain; 10 Departament de Matemàtiques, Universitat Autònoma de Barcelona, Barcelona, Spain

**Keywords:** CAR-T cells, CAR-NK cells, mitochondria, BH3 mimetics, BH3 profiling

## Abstract

Therapy-induced senescence (TIS) generates an immunogenic state in cancer cells by altering how they present antigens, produce cytokines, and organize their surfaceome. TIS can be exploited for therapeutic purposes using “immunosenolytic” strategies, including adoptive cellular therapies such as chimeric antigen receptor (CAR)-engineered T and natural killer (NK) cells. A frequently overlooked barrier may limit the success of these living drugs: mitochondrial apoptotic priming in the target TIS cancer cells. Contrary to the prevailing dogma, recent assessments of mitochondrial apoptotic signaling via BH3 profiling (a functional assay measuring proximity to the mitochondrial apoptotic threshold and identifying BCL-2 family dependencies) have revealed that TIS cancer cells are globally less primed for apoptosis than their proliferating precursors. TIS cancer cells exhibit a conserved, druggable dependence on specific members of the BCL-2 family for survival. Interestingly, the pre-existing priming and anti-apoptotic addictions of parental, non-senescent cells, are retained upon induction of senescence. This suggests an “inherited” mitochondrial memory that may predict the (immuno)senolytic responsiveness of TIS cancer cells. BH3 profiling could help to personalize CAR-based immunosenolytic therapy according to apoptotic readiness across pre- and post-TIS states. This companion diagnostic could inform the rational use of BH3 mimetics in combination with CARs and guide the engineering of precision immunosenolytic interventions such as “armored” CAR-T/NK cells neutralizing specific anti-apoptotic dependencies at the effector-target interface. This perspective reframes mitochondria as predictive checkpoints that can be monitored and targeted to enable TIS cancer cells to respond precisely and durably to adoptive CAR-T/NK immunotherapy within “one-two punch” senogenic-immunosenolytic designs.

## 1 Introduction

**Figure 1:**
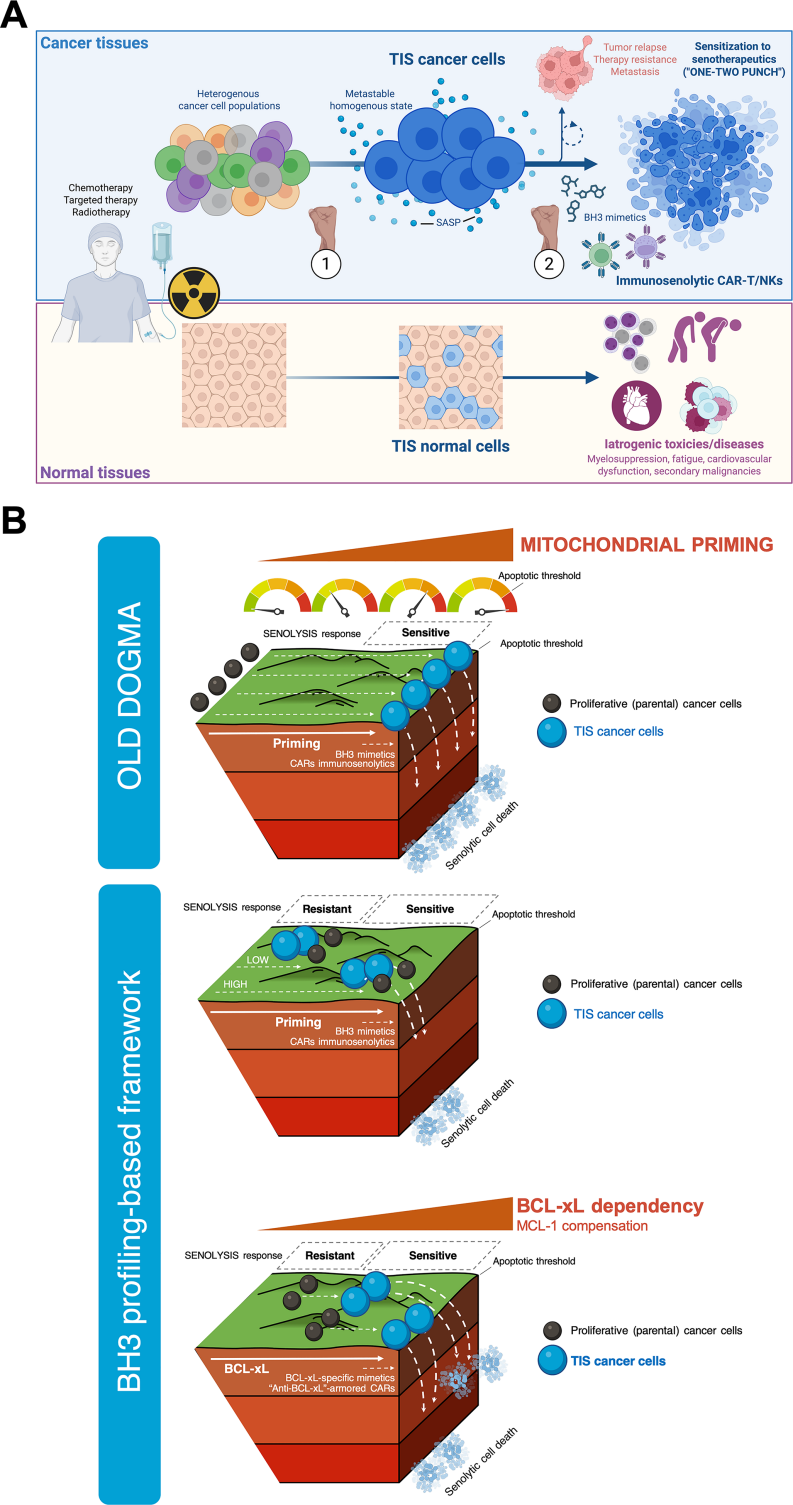
**(A)** Therapy-induced senescence (TIS) as a therapeutic gateway: opportunities and risks of one–two punch senogenic-(immuno)senolysis in cancer and normal tissues. Senogenic therapies such as radiotherapy, certain cytotoxics, and CDK4/6/PARP inhibitors (“punch 1”) can promote a common, metastable senescent state (TIS) among heterogeneous cancer cell subpopulations. This strategy effectively “levels the playing field” by creating common sensitivities to subsequent senolytics or immuno-senolytics targeting the specific vulnerabilities shared by TIS cancer cells. The TIS phenomenon significantly rewires antigen presentation, SASP/IFN signaling, and the surfaceome composition, generating tractable liabilities for selective (immuno)senolysis. The “one-two punch” schema aims to exploit this window by delivering a timed senolytic hit (“punch 2”) –small-molecule BH3 mimetics or adoptive cellular “immunosenolytics” such as CAR-T/NK targeting specific TIS senoantigens– which is expected to eradicate relapse-prone TIS reservoirs, preempting minimal residual disease, therapeutic resistance, and metastasis. In tumor tissues, successful (immuno)senolysis converts the immunogenic, yet apoptosis-buffered, TIS state into irreversible mitochondrial death. In normal tissues, controlled senolysis mitigates the adverse effects of therapy-provoked senescent cells, including myelosuppression, fatigue, cardiovascular dysfunction, and even secondary malignancies. TIS is therefore a tunable, immunogenic opportunity that, when paired with precisely timed pharmacological or cellular immunosenolysis, can increase efficacy in both cancerous and normal tissues. Strategic TIS antigen selection and logic-gated CAR designs aim to confine immunosenolysis to TIS compartments while preserving healthy tissues. **(B)** Mitochondrial priming and anti-apoptotic addictions in TIS cancer cells: Revisiting the old dogma to move forward. This conceptual framework integrates recent BH3 profiling studies across multiple senescence models, revealing a unifying mitochondrial adaptation. It has historically been assumed that TIS cancer cells are uniformly “apoptosis-prone,” hyper-primed for mitochondrial death overall, and susceptible to any pro-apoptotic BH3 mimetic or immune effector cytotoxicity. According to this dogma, TIS is a universal sensitizer to cell death by (immuno)senolytics (top panel). However, recent functional analyses [70–73] challenge this perspective. These analyses demonstrate that TIS cancer cells are less primed for apoptosis than their proliferating counterparts. This explains their broad refractoriness to cytotoxics and their highly variable responsiveness to pro-apoptotic BH3 mimetics (middle panel). Importantly, despite reduced overall priming, TIS cancer cells consistently rely on BCL-xL for survival. This is evident from their increased sensitivity to HRK or BAD peptides and to BCL-xL-selective antagonists (bottom panel). Mechanistically, BCL-xL neutralizes stress-adapted pro-apoptotic effectors by sequestering BAK and redistributing BAX to the cytosol, thereby blocking MOMP. HRK downregulation further frees BCL-xL to reinforce BAK binding. This explains why selective BCL-xL inhibition restores priming competence. The apoptotic priming state and anti-apoptotic blockades of parental, non-senescent cells foreshadow senolytic susceptibility, revealing an “inherited” mitochondrial memory that predicts responsiveness to specific BH3 mimetics. The figure illustrates the spectrum of TIS phenotypes, which are defined by variable priming and differential reliance on anti-apoptotic BCL-2 proteins such as BCL-xL. However, resistance can emerge through compensatory MCL-1 upregulation, underscoring the need for dynamic BH3 profiling and rational combination therapy. Together, these findings redefine TIS as a reprogrammable state with a conserved, druggable anti-apoptotic bottleneck that can be exploited through BH3 profiling-guided pharmacological or immunological senolysis, rather than as an inert, apoptosis-resistant endpoint.

Therapy-induced senescence (TIS) of cancer and normal cells is a collateral outcome of radiotherapy, DNA-damaging chemotherapy, and several targeted agents, including CDK4/6 inhibitors (e.g., palbociclib) and PARP inhibitors (e.g., olaparib). The “one-two punch” senogenic-senolytic strategy, pioneered by René Bernards (1–4), aims to exploit the TIS phenomenon clinically. This strategy may represent a paradigm shift in preventing cancer recurrence and limiting the systemic side effects of cancer therapies (5–10; **Figure 1A**). The “first punch” involves using a senogenic agent to induce a deep, targetable senescent state in tumor cells. This primes them for subsequent elimination by a second punch with a senotherapeutic agent that exploits 0vulnerabilities unique to the senescent phenotype. The selective elimination of TIS cells within cancer tissues is expected to prevent the development of drug resistance, tumor recurrence, and metastasis. The elimination of TIS cells in normal cells is expected to mitigate treatment-related, iatrogenic sequelae such as myelosuppression, organ fibrosis, inflammation, frailty and fatigue (5–7). The implications of the senogenic-senolytic strategy extend beyond oncology, offering potential interventions for fibrosis, neurodegeneration, and other age-related diseases in which the accumulation of senescent cells is pathogenic (8–16).

The “one-two punch” approach aims to “*level the playing field*” in initially heterogeneous cancer cell populations by creating a more homogeneous TIS population with a common sensitivity to synthetic lethal interactions through sequential, tailored use of senolytics or immunosenolytics (**Figure 1A**). This strategy provides an updated, “2.0” version of the traditional synthetic lethality approach by separating the timing of senescence induction and senotherapy to maximize therapeutic efficacy and minimize combinatorial toxicity typical of concurrent, vertical pathway targeting in cancer therapy (1–4, 17–22). However, the first generation of small-molecule senolytics, which are mostly pro-apoptotic BH3 mimetics, has faced significant challenges limiting their potential for translation. These challenges include undesirable side effects and, particularly, a highly variable TIS cancer cell sensitivity, ranging from high responsiveness to complete refractoriness (23–29). This may necessitate the development of companion diagnostics to predict how TIS cancer cells will respond to personalized senolytic drugs. Alternatively, one promising way to maximize the specificity, potency, and clinical safety of senotherapeutics is to exploit immunotherapy to target senescence-specific surface antigens. In particular, T cells and natural killer (NK) cells that are redirected with a chimeric antigen receptor (CAR) to target proteins uniquely expressed on TIS cancer cell surfaces are being actively explored as “living” immunosenolytics (30–34). These synthetic immune cells can selectively and effectively eliminate TIS cancer cells due to their cytotoxic potency, effective migratory capacity, ability to self-expand, and memory capabilities. Most of the efforts in this area focus on avoiding side effects in healthy, non-cancerous tissues by identifying antigens unique to cancer cell senescence (senoantigens) to target subpopulations of TIS cancer cells with high specificity. However, the intrinsic resistance of TIS cancer cells to the apoptotic stimuli triggered by CAR-T/NK cells is an often-overlooked barrier that may limit the success of these living drugs but has received very little attention.

In this review, we first provide a brief evaluation of the rationale behind using CAR-T/NK cells as living immuno-senolytics. Next, we discuss recent studies that address the paradox of how TIS cancer cells are refractory to most apoptosis-inducing therapeutics yet are variably sensitive to apoptosis-inducing BH3 senolytics. Finally, we explore how this knowledge can inform the successful therapeutic application of TIS-targeting CAR-T/NK cells as part of “one-two punch” senogenic-senolytic strategies.

## 2 Therapy-induced senescence: from tumor suppression to an immuno-oncology strategy

TIS-induced irreversible proliferative arrest is considered a *bona fide* tumor-suppressive program. However, the senescence-associated secretory phenotype (SASP) can promote the persistence and survival of TIS cancer cells by enabling them to evade immune clearance. Conversely, immune cells, including CD8^+^ T cells and NK cells, can naturally eliminate senescent cells. Therefore, certain TIS agents can promote tumor regression by stimulating immune-mediated senolysis. Reactivating of senescence programs in cancer cells is an underappreciated aspect of immuno-oncology that can make tumors more susceptible to immunotherapy, particularly cellular immunotherapy (21, 22, 35–37).

The first proof of principle that cancer cell senescence is an immunogenic cell state rather than a mere cytostatic “end” came from demonstrating that reactivating p53 in Ras/Myc-driven murine liver carcinomas induces senescence in tumor cells. This triggers a CSF1- and CCL2-rich SASP that recruits polymorphonuclear leukocytes and macrophages. This archetypal “senescence surveillance” ultimately results in a rapid, perforin-dependent tumor clearance (38). Fifteen years later, a systematic analysis of the anti-tumoral adaptive immune response elicited by the TIS phenomenon was performed using a Myc-driven lymphoma model. In this model, TIS induced by chemotherapy and CDK4/6 inhibitors was found to be highly immunogenic. TIS cancer cells upregulate the antigen-processing machinery and expand the MHC-I peptidome. They also secrete a SASP dominated by type I and II interferons (IFNs), which elicits robust, protective, tumor-specific CD8^+^ T-cell responses. Genetic or pharmacological inhibition of STING, β2-microglobulin, or perforin neutralizes this effect, thereby placing senescent cells upstream of adaptive antitumor immunity (39).

We now know that the senescence programs in cancer tissues are not static from an immunological perspective. A striking remodeling of the “surfaceome” occurs in the senescent cells of chemically induced liver cancers. This remodeling process involves increased IFNγ receptors, adhesion molecules, and TNF-death receptors, as well as decreased “*don’t eat me*” signaling surface molecules, such as CD47. These changes drastically alter how a TIS-enriched tumor senses and responds to environmental cues. Importantly, these changes amplify STAT1 signaling and create a feed-forward loop between the senescence surfaceome and adaptive immunity. This enables CD8^+^ T cells and NK cells to target TIS cancer cells more efficiently (40).

These pioneering studies have broadened our understanding of the immune surveillance of senescent cancer cells by extending it beyond innate sensors (41), which have also been shown to induce CD8^+^ T-cell infiltration and tumor control in response to TIS. DNA damage, induced by irradiation or CDK4/6 inhibition, drives the accumulation of cytosolic DNA, activating cGAS-STING signaling, a key component of the innate immune system in the settings of infection, cellular stress, and tissue damage. This elicits a type I IFN response, connecting innate and adaptive immunity and ultimately upregulating the antigen-presenting machinery in TIS cancer cells (42–45). Senescent lymphoma cells have been shown to even undergo further immunogenic changes. Accompanying chromatin and lineage reprogramming engenders a dendritic cell-like state that directly boosts T cell-mediated clearance and predicts superior survival in diffuse large B-cell lymphoma patients (46).

Taken together, these studies strongly support exploring new immunotherapy combinations based on the TIS phenomenon and guided by three key principles. First, TIS cancer cells can exert cell-extrinsic cytotoxicity by regulating the immune system. Second, the TIS-related immune effects are SASP-mediated. Third, immune cells alone can suffice to reduce the size of TIS-enriched tumors. Rather than viewing TIS as a passive byproduct of cancer therapy, it should be viewed as a tunable immunogenic rheostat that can either sensitize or insulate tumors from modern immunotherapeutic modalities.

## 3 Selective and efficient elimination of TIS cancer cells: immunotherapies with senolytic activity

The evidence presented above establishes the conceptual and mechanistic basis for deliberately triggering senescence using genotoxic, cytostatic, or targeted agents to stimulate or enhance the ensuing of innate and adaptive immune responses against tumors. Eliminating the immunosuppressive effects of the TIS phenomenon while leveraging its immunogenic functions is a coherent approach to broadening the efficacy of immunotherapy (47, 48).

Senomorphics, a class of senotherapeutics that restrain the pro-inflammatory activity of senescent cells and/or attenuate the detrimental effects of SASP components (4, 49–51), could minimize the paracrine-mediated immunosuppressive effects of SASP without causing the death of TIS cancer cells. Examples of senomorphics include rapamycin, metformin, resveratrol, and aspirin. However, information about the SASP-related capacity of senomorphics to enhance the natural or engineered immunosenolysis of TIS cancer cells is currently limited. TIS cancer cells can also establish a cell-autonomous immune barrier that restricts the activity of cytotoxic T-cells. They can accomplish this by transcriptionally activating the expression of the immune checkpoints PD-L1 and PD-L2, as well as post-translationally enhancing their stability (e.g., via glycodecoration) on the cancer cell membrane (52–57). Overactivation of PD-1/PD-L2 has been observed across various aging tissues and TIS models. While this overactivation is not necessary for cancer cells to undergo senescence, it is essential for senescent cancer cells to evade the immune system and persist in tumors. These observations highlight the conserved yet targetable PD-1:PD-L1/2 axis of immune evasion arising from the TIS phenomenon. The growing number of FDA-approved and experimental drugs targeting PD-1, PD-L1, and PD-L2 suggests that immuno-senolytic strategies promoting tumor regression via CD8+ T cells and cancer therapies inducing senescence will be developed in the near future.

In addition to developing antibody-drug conjugates (ADCs), antigen-conjugate senolytic vaccines, and senolytic bispecific T-cell engagers (BiTEs) that target senescence-specific surface antigens (e.g., β2-microglobulin), vaccination with TIS cancer cells has been shown to be a more efficient method of inducing immune system protection against tumor relapse than immunogenic cell death (16, 39, 58, 59). Significant protection from tumor engraftment and tumor regression, as well as enhanced immunotherapy efficacy, has been observed upon injection of senescent cancer cells or dendritic cells activated by senescent cells (60). Normal senescent cells can also be used for anti-cancer immunization because altering their immunopeptidome activates CD8^+^ T lymphocytes (61). Similarly, the generation, purification, and injection of senescent cancer cell-derived nanovesicles (SCCNVs), which present a wide variety of specific antigens, can act as natural immune adjuvants (62). These adjuvants can improve vaccine immunogenicity, activate the CD8^+^ T cell population, and slow tumor progression.

Nevertheless, the most rapidly advancing strategy is the therapeutic use of adoptive cellular immunotherapy against TIS cancer cells. Researchers have demonstrated that engineered chimeric antigen receptor (CAR) T and NK cells can function as “live” immunosenolytic agents that selectively and effectively eliminate TIS cancer cells. The urokinase-type plasminogen activator receptor (uPAR) is a cell-surface protein that is widely expressed in senescent cells, but expressed at relatively low levels in normal tissues. uPAR-specific CAR-T cells have been shown to clear TIS cancer cells and extend survival remarkably in mice with lung cancer treated with senescence-inducing drugs (30, 63). Senescence also decorates the plasma membrane with stress-induced natural killer group 2 (NKG2D) ligands (MICA, MICB, and ULBP1–5), thus creating a tractable target for engineered senolytic immune cells (64, 65). While not formally tested in TIS cancer cells, NKG2D-CAR T cells have demonstrated robust cytotoxicity against senescent mouse embryonic fibroblasts and mouse forebrain astrocytes that have been induced by DNA damage and oxidative stress (32, 33).

Together, these proof-of-concept studies strongly support the therapeutic potential of using senolytic CAR-T/NK cells to treat senescence-associated diseases and to eradicate therapy-persisting senescent cancer cells with enhanced precision as part of “one-two punch” senogenic-immunosenolytic strategies.

## 4 Using CAR-T/NK as living senolytics against TIS cancer cells: promises and challenges

Leveraging the specificity, potency, and clinical safety of CAR-T/NK cells to treat TIS-enriched tumors is appealing (31, 66–69). Unsurprisingly, CAR-T and CAR-NK cells are rapidly emerging as the next generation of immunity-based senotherapies. CAR-T/NK cells can eradicate therapy-persisting TIS cancer cells due to features that distinguish them apart from traditional approaches. First, they have an intrinsic specificity based on the recognition of senescence-associated surface antigens, a capability that surpasses current small-molecule approaches. Current small-molecule senolytic approaches (e.g., pro-apoptotic BH3 mimetics) vary widely in their senolytic activity against TIS cancer cells. These approaches range from highly active to completely inactive and are commonly accompanied by undesirable off-target effects (70–73). Second, CAR-T/NK cells are living drugs that can exponentially expand and kill hundreds or thousands of target TIS cancer cells. Conversely, they can contract when the target senescence-associated antigen is no longer present, remaining on patrol for years (30, 31, 74, 75).

The infusion dose of adoptive CAR-T/NK cells is important for addressing safety concerns, reducing exhaustion, and optimizing cytotoxic efficiency (68, 76, 77). However, the therapeutic index of CAR-T/NK cells is an important yet largely unexplored area of study. The success of synthetic CAR-T/NK cells is believed to depend on their use of the highly efficient endogenous pathway underlying T/NK cell toxicity. Improving the initial CAR T cell-directed killing capacity of cancer cells would make limitations to successful CAR T cell therapy, such as the selection of antigen-negative cancer cells and failed CAR T cell expansion and persistence, less relevant (78).

Due to the ample evidence that CAR T cell-induced apoptosis is often suboptimal and that cancer cell resistance to apoptosis limits effective responses to CAR T cell therapy, there are increasing attempts to improve their overall killing capacity. Some platforms, such as TRUCKs (T cells redirected for universal cytokine-mediated killing) and SEAKERs (synthetic enzyme-armed killer cells), aim to remodel the tumor microenvironment to indirectly increase the priming of cancer cells for apoptosis. TRUCKs secrete pro-inflammatory cytokines, such as IL-12 or IL-18 (79), while SEAKERs deliver small-molecule pro-drugs (80, 81). More recent strategies focus on equipping CAR T cells with pro-apoptotic modules that aim to directly augment the apoptotic priming of target cancer cells by neutralizing survival pathways (82–85). One prominent example is the granzyme B-NOXA fusion protein, which delivers the BH3-only protein via the perforin-granzyme axis and selectively antagonizes the anti-apoptotic MCL-1 protein in target cancer cells (86). This targeted delivery method avoids the systemic toxicity associated with pharmacological MCL-1 inhibitors, yet maintains CAR T persistence and spares non-malignant tissues. Another approach exploits sensitization for extrinsic apoptosis via the death receptor TRAIL-R2 and downstream signaling molecules, such as FADD, BID, and caspase-8, thereby reinforcing CAR T–triggered cytotoxicity. More broadly, modular “armored” CAR T designs can incorporate other BH3-only pro-apoptotic proteins (e.g., BIM or PUMA) or death receptor agonists to overcome tumor-specific blocks in apoptosis activation. Precision engineering that integrates these pro-apoptotic modules with TRUCK-based cytokine delivery or SEAKER enzyme payloads could provide a tailored, multi-pronged approach to enforcing apoptotic commitment and achieving a deeper, relapse-resistant CAR T cell response.

We recently learned that mitochondrial apoptosis in cancer cells is necessary for NK/T cell cytotoxicity. The mitochondrial outer-membrane permeabilization (MOMP) phenomenon is the critical barrier that determines cytolytic responses at the low effector-to-target ratios typical of clinical CAR-T/NK cell infusions (87). Genetic ablation of the apoptotic effectors BAX and BAK in cancer cells results in the loss of MOMP. This loss is sufficient to almost completely abrogate NK/T cell-mediated cell death when physiologically relevant ratios of ≤ 1 (effector) NK cell to 1 (target) cancer cell are present. However, killing can still occur at supraphysiological ratios if “strong-killer” NK populations are exceptionally cytotoxic. Importantly, this rate-limiting mitochondrial gateway can be pharmacologically primed.

BH3 profiling is a functional barometer that determines how close a cell is to the mitochondrial apoptotic threshold. It assesses mitochondrial susceptibility to MOMP in response to synthetic peptides that encode the active domains of pro-apoptotic BH3 proteins, such as BIM, BID, BAD, PUMA, NOXA, MS1, FS1, BMF, and HRK. These proteins interact promiscuously or selectively with pro-survival BCL-2 family proteins. A BH3 profiling assay determines the overall state of mitochondrial priming, also known as the “readiness” to undergo apoptosis, and identifies specific dependence on one or more anti-apoptotic proteins, such as BCL-2, BCL-xL, BCL-w, MCL-1, and BFL-1. This process is also known as “apoptotic blockade” (88–99). BH3 profiling revealed that brief, sublethal synapses with NK/T immune cells cumulatively elevate mitochondrial priming and alter anti-apoptotic dependencies in survival cells without triggering cell death. These changes were observed as early as two hours after culturing the effector and target cells, suggesting that serial hits by NK cells incrementally bring the mitochondrial rheostat of survivors closer to the apoptotic threshold. Conversely, the overexpression of anti-apoptotic proteins such as BCL-2, BCL-xL, and MCL-1, decreases mitochondrial priming and proportionally reduces NK/T cell cytotoxicity. These findings underscore the notion that the outcome of an NK/T effector synapse is ultimately determined by the intrinsic buffering capacity of the target cancer cell’s mitochondria (87).

When IL-2-activated primary NK cells were combined with BH3 mimetics that matched or mismatched the BH3 profiling–defined anti-apoptotic dominant dependencies of a panel of tumor cell lines, only the matched combinations yielded significant synergy (e.g., BCL2 blockade in BCL2-addicted acute-myeloid leukemia or MCL-1 blockade in MCL-1-dependent cervical carcinoma cells). Non-matched inhibitors, on the other hand, remained inert. Importantly, treatment with a combination of pre-activated NK cells and matched BH3 mimetics increases mitochondrial priming *ex vivo*, reduces tumor burden, and extends median survival in NSG xenografts by approximately two weeks compared to monotherapy. However, mismatched regimens failed to provide additional benefits. Thus, the *in vivo* data corroborate the idea that the readiness of the mitochondrial in target cancer cells, rather than the quality of the immune effector cells, dominates the variance in the response to CAR-T/NK cells. Nevertheless, pre-activated NK cells themselves exhibit low mitochondrial priming and heightened anti-apoptotic reserves themselves, conferring over 100-fold resistance to BH3 mimetics targeting BCL-2, BCL-xL, and MCL-1 (87). This provides a non-overlapping therapeutic window that could be exploited to preserve effector fitness.

Both indirect strategies, such as TRUCKs and SEAKERs, and direct strategies, such as armoring CARs with an apoptotic cargo, converge on the notion that they will reduce primary resistance in cancer cells and lower the likelihood of disease relapse by increasing the priming of target cancer cells for apoptosis. Mitochondrial priming and specific apoptotic blockades can determine the immune cytolysis response of cancer cells regardless of the T/NK cell source (primary, cell line, or engineered). Together, this information can be used to optimize and personalize CAR-T/NK cells as (immuno)senolytics in “one-two punch” clinical strategies that aim to exploit the TIS phenomenon. In this scenario, adoptive T/NK-based immunosenolytic therapy can be considered a two-component system. Engineered immune cells deliver the first component: the senolytic signal. This signal must be matched by the second component, a receptive mitochondrial circuit, in target TIS cancer cells. A functional readout based on BH3 profiling could match TIS cancer cells with the optimal CAR-T/NK immunosenolytic. This could involve combining currently existing CAR-T/NK products combined with tailored BH3 mimetics, or developing next-generation CAR-T/NK cells armored with specific pro-apoptotic agonists. This approach aligns with precision oncology paradigms and could accelerate the design of “one-two punch” senogenic-immunosenolytic clinical trials.

However, contrary to the accepted prevailing dogma in the field, we have recently learned that TIS cancer cells generally exhibit a lower level of overall apoptotic priming than their parental cells but appear to share a pro-survival adaptation to BCL-xL (**Figure 1B**). Indeed, the variability in sensitivity to apoptosis-inducing BH3 senolytics observed in TIS cancer cells could be explained by the mitochondrial circuitry state of non-senescent parental cells (70–73). This underscores the urgent need to better understand how the mechanistic basis of the variable response to small-molecule BH3 drugs can also influence the response to CAR-T/NK immunosenolytics.

## 5 BH3 profiling: dissecting the dependence of TIS cancer cells on mitochondrial priming

Four recent studies from the Letai, Bernards, Montero, and Menendez groups address the paradox that TIS cancer cells are resistant to common cancer therapeutics that induce apoptosis yet are variably sensitive to pro-apoptotic BH3 senolytics (**Figure 1B**) (70–73). These studies test the hypothesis that variability in mitochondrial priming overall, or proximity to the apoptotic threshold, and/or dependence on specific anti-apoptotic BCL2 family members underlies this inconstant sensitivity. Assuming that the abundance of anti-apoptotic BCL-2 proteins in TIS cells and the dynamic evolution of the BCL-2/BH3 interactome at the mitochondria are critical to determining heterogeneous responses to BH3 senolytic drugs, the researchers used different versions of the so-called BH3 profiling to predict TIS cancer cell sensitivity to these drugs.

The common assumption that senescent cells are resistant to apoptosis is largely based on the correlative observation that they generally respond less to various cytotoxics than proliferating cells do. However, it has not been properly tested whether a general reduction in drug sensitivity is a feature specifically acquired by the senescent state in paired models of senescent and non-senescent parental cells. MacDonald et al. (70) addressed this challenge by testing how various senescence triggers (i.e., replicative senescence, oncogene-induced senescence, and doxorubicin-induced senescence) affect the response of diverse non-cancerous and cancerous cells to (1) cytotoxic chemotherapies, (2) BH3 mimetics, and (3) other reported senolytics (e.g., fisetin, dasatinib, and quercetin). The resistance of senescent cells to these drug treatments, compared to that of proliferating parental cells, depended on the drug class and the specific drug studied within each class, and it was highly heterogeneous. Among cytotoxic drugs, senescent cells were more resistant to alisertib (a selective Aurora A kinase inhibitor), etoposide (a topoisomerase II inhibitor), and staurosporine (a protein kinase C inhibitor) but not to doxorubicin (a DNA intercalator and topoisomerase II inhibitor). Among BH3 mimetics, senescent cells were more sensitive to mimetics that target BCL-xL such as ABT-263/navitoclax and A1331852. However, they remained indifferent to the BCL-2 inhibitor ABT-199 and the MCL-1 inhibitor S63845. Among non-BH3 senolytics, senescent cells were more sensitive to dasatinib (D) than to quercetin (Q) or the D+Q combination. Therefore, although senescent cells may be resistant to chemotherapeutic agents, they are highly responsive to pro-apoptotic senolytics that target BCL-xL.

In the search for phenotypic and molecular features associated with the acquired susceptibility of senescent cells to specific senolytics, traditional markers of senescence were not predictive of senolytic efficacy. These markers included senescence-associated (SA)-β-gal positivity, cell cycle arrest drivers, transcriptional signatures of apoptosis-related genes, and components of the SASP (100–102), with the exception of high *CXCL8* expression. Unexpectedly, senescent cells were found to have mitochondria that are less primed for apoptosis, as was previously observed in cancer cells that are resistant to cytotoxic chemotherapy and BH3 mimetics (95, 103, 104). This finding provides a physiological basis for the resistance of senescent cells to cytotoxic chemotherapeutic drugs. However, how can this be reconciled with their increased sensitivity to specific pro-apoptotic BH3 mimetics? A key finding of the MacDonald et al.’s study (70) is the observed strong correlation observed between the cytochrome c release occurring upon HRK peptide exposure and sensitivity to ABT-263/navitoclax and the D +Q combination in senescent cells. Since the HRK BH3 peptide selectively targets BCL-xL and provides a functional readout of BCL-xL dependence in apoptosis regulation, these data suggest that BCL-xL is a common and targetable vulnerability of the senescent state. Senescent cell mitochondria have two characteristics that explain why they are refractory to apoptosis-inducing therapeutics yet sensitive to apoptosis-inducing BH3 mimetics: (1) a reduced overall mitochondrial priming compared to proliferating cells, which explains their reduced sensitivity to apoptosis-inducing cytotoxic drugs, and (2) an increased dependence on BCL-xL for survival. This dependence makes them sensitive to BCL-xL inhibitors, such as ABT-263/navitoclax and A1331852, as well as to D+Q, since quercetin weakly antagonizes BCL-xL/BCL-2 (105). Therefore, BCL-xL inhibition could be considered as a functional mechanism of senolysis that can be successfully predicted by BH3 profiling, even in the absence of corresponding transcriptional or translational upregulation of *BCL-xL* mRNA or BCL-xL protein.

### 5.1 Intrinsic mitochondrial dependencies in the non-senescent state influence the senolytic sensitivity of senescent cancer cells to ABT-263/navitoclax

These findings challenge the oversimplified classification of senescent cells as “apoptosis-resistant” and the broad classification of BH3 drugs and other BH3-like compounds as universally senolytic (**Figure 1B**). They also highlight the importance of assessing mitochondrial dependencies, such as HRK peptide sensitivity via BH3 profiling, rather than broad molecular markers of senescence, when identifying predictive functional biomarkers that predict the efficacy of senolytics in one-two punch strategies. To formally test this hypothesis, Jochems et al. (71) systematically investigated the mitochondrial priming of TIS cancer cells and their sensitivity to ABT-263/navitoclax using BH3 profiling in a broad panel of cancer cell lines. TIS cancer cells generated by mechanistically distinct senescence triggers (e.g., alisertib, etoposide, PF-06873600 [a CDK2/4/6 inhibitor], and ionizing radiation) exhibited a reduced overall mitochondrial priming as measured by BIM-induced cytochrome c release, compared to their parental state. This finding confirms that the widely accepted notion that senescent cells are primed for apoptosis cells is no longer valid. Interestingly, the apoptotic priming state of the (pre-senescent) parental cells predicted the senolytic sensitivity of their senescent (TIS) counterparts to ABT-263/navitoclax. Cancer cells with high mitochondrial apoptotic priming in the non-senescent state –as indicated by a higher response to BIM or PUMA, the two major pro-apoptotic antagonists of BCL-xL– are predisposed to ABT-263/navitoclax sensitivity upon senescence induction. This effect is independent of the senescence inducer. This finding reveals the unexpected result that pre-existing anti-apoptotic mitochondrial dependencies in pre-senescent cancer cells (e.g., BCL-xL) are retained upon senescence induction. Consequently, despite an overall decrease in apoptotic priming compared to their parental state, TIS cancer cells exhibit selective dependence on particular anti-apoptotic BCL-2 family members (**Figure 1B**). ABT-263/navitoclax-sensitive TIS cancer cells exhibit higher mitochondrial cytochrome c release in response to BIM and PUMA peptides, confirming their “inherited” increased dependence on BCL-xL. Conversely, ABT-263/navitoclax-resistant TIS cancer cells show a compensatory MCL-1 upregulation, serving as a resistance mechanism against ABT-263/navitoclax-induced apoptosis (71). This may explain the lack of a strong correlation between HRK-mediated cytochrome c release and ABT-263/navitoclax sensitivity. These results suggest that additional anti-apoptotic adaptations may buffer against BCL-xL inhibition. Consistent with this idea, dynamic BH3 profiling following ABT-263/navitoclax treatment further demonstrates a shift in dependency from BCL-xL to MCL-1 in sensitive cells (31). While BCL-xL inhibition is necessary for senolysis, it is not always sufficient to define senolytic susceptibility, especially when BAX/BAK is deficient. These findings underscore the importance of incorporating functional BH3 profiling to identify the presence of broader, compensatory apoptotic pathways in TIS cancer cells.

### 5.2 BCL-xL mediates senolytic resistance of TIS cancer cells via HRK downregulation and BAK binding

A key mechanistic finding from the study by Jochems et al. (71) is that mitochondrial BAX is lost in TIS cancer cells when the cell membrane is permeabilized during BH3 profiling, while BAK remains unaffected. This effect is related to BCL-xL’s ability to shuttle BAX between the mitochondria and the cytosol in a stress-dependent manner. This mechanism may serve as an adaptive survival strategy in TIS cancer cells. In cancer cells with low stress levels (e.g., proliferating cells or cells in a shallow, transient senescent state), BCL-xL predominantly sequesters the activator proteins BIM and BID. In contrast, in cancer cells with elevated stress levels (e.g., cells in a deep, stable senescent state), BCL-xL binds to the effector proteins BAK and BAX. This leads the translocation of BAX to the cytosol (106, 107). This redistribution of BAX to the cytosol, driven by senescence stress and BCL-xL, may impair HRK-induced MOMP. This explains the altered response of TIS cancer cells to the HRK peptide and their susceptibility to ABT-263/A1331852, which restores apoptotic competence by disrupting the BCL-xL/BAX interaction.

Alcon et al. (72) investigated the nature of the apoptotic priming of TIS melanoma cells and found that a BCL-xL-mediated survival mechanism conferred apoptotic resistance. TIS melanoma cells exhibited variable apoptotic signatures, including BIM downregulation, variable BAX expression, and stable or even increased BAK expression, across cell lines. However, BH3 profiling revealed consistent BCL-xL pro-survival adaptations, evidenced by an increased cytochrome c release in response to BCL-xL BH3 peptide antagonists such as HRK and BAD. Regardless of the senescence trigger (the CDK4/6 inhibitor palbociclib or γ-irradiation), downregulation of HRK was found to be central to the apoptotic signature of the TIS phenotype. This increases the availability of BCL-xL, which tightly binds and sequesters BAK, thus preventing MOMP and apoptosis. The Alcon et al. study demonstrates that targeting BCL-xL with ABT-263/navitoclax, A1331852, or a selective BCL-xL PROTAC degrader (108–112) effectively eliminates TIS melanoma cells. In contrast, specific inhibitors targeting BCL2- or MCL-1 show limited efficacy (72). The mechanistic and therapeutic implications are twofold: (1) BCL-xL is a primary anti-apoptotic barrier in TIS cancer cells, and HRK downregulation is a molecular driver that can be targeted to eliminate TIS cancer cells; and (2) strategies that prevent HRK loss or disrupt the BCL-xL-BAK interaction directly may enhance the efficacy of senolytics against TIS cancer cells.

### 5.3 BH3 profiling can predict BCL-xL dependency and the response to senolytics of TIS cancer cells

The study by Alcon et al. (72) highlights the translational relevance of functional BH3 profiling as a predictive biomarker for more accurate senolytic response and senolytic drug selection, rather than transcriptional or proteomic analysis of the BCL-2:BH3 interactome components. López et al. (73) further emphasized the need for BH3 profiling to guide the selection of senolytic strategy by examining mitochondrial priming states and apoptotic dependencies in a diverse panel of TIS models with different genetic backgrounds (e.g., *p16*-null/*p53*-proficient, *BAX*-deficient, and *BRCA1*-deficient), which were induced by mechanistically different agents including palbociclib, alisertib, doxorubicin, bleomycin (an oxidative DNA strand scissor), and olaparib (a poly(ADP-ribose) polymerase inhibitor). They found that increased mitochondrial priming correlates with increased sensitivity to the BCL-xL-targeting BH3 mimetics, such as ABT-263/navitoclax and A1331852. However, increased mitochondrial priming was not a universal feature of all TIS phenotypes or a strict requirement for BCL-xL-targeted senolysis. Rather, BCL-xL emerged as a conserved anti-apoptotic determinant across different TIS models, including those lacking increased mitochondrial priming. BH3 profiling with sensitizing peptides that preferentially disrupt BCL-xL interactions (e.g., HRK) functionally confirmed the strong dependence on BCL-xL as a dominant survival factor that maintains mitochondrial integrity even in minimally primed TIS cancer cells. This dependence was also validated by the significant sensitivity to the BCL-xL-selective BH3 mimetic A1331852 in multiple TIS contexts, including both stable and transient senescence. TIS cancer cells with low or moderate priming and an unstable senescent phenotype [e.g., those induced by palbociclib or olaparib (113–116)] still show marked responsiveness to A-1331852. These results highlight the robustness of BCL-xL as a critical regulator of apoptosis in TIS and suggest that selective BCL-xL inhibition may be an effective therapeutic strategy regardless of the priming state of TIS cancer cells. The mechanistic and therapeutic implications are threefold: (1) BCL-xL dependence appears to be a universal feature of TIS, independent of mitochondrial priming or TIS stability; (2) BH3 profiling (e.g., HRK-mediated depolarization) can predict a BCL-xL-targeted senolytic response; and (3) stable TIS phenotypes respond to both BCL-xL/BCL-2 dual inhibitors (ABT-263/navitoclax) and BCL-xL-selective drugs (A1331852), whereas transient TIS phenotypes respond only to selective BCL-xL inhibition.

A central finding is that the TIS phenomenon can be viewed as a spectrum of cellular states with varying levels of apoptotic readiness, BCL-xL dependency, and senolytic responsiveness (73). This continuum ranges from a shallow, transient senescence to deep, stable senescent arrest, which may be pre-determined by the mitochondrial priming state and apoptotic dependencies of the parental cancer cells and/or the tissue of origin (71). Shallow/transient TIS phenotypes are characterized by minimal mitochondrial priming, a tendency to regain proliferative capacity, moderate sensitivity to BCL-xL inhibition with A1331852, and limited response to the broad-spectrum drug ABT-263/navitoclax. These phenotypes also exhibit BCL-xL dependence that is decoupled from overt mitochondrial priming. In these phenotypes, the TIS maintenance mechanisms are more critical than apoptotic readiness. Deep/stable TIS phenotypes exhibit strong mitochondrial priming and robust dependence on BCL-x. These phenotypes are highly sensitive to both ABT-263/navitoclax and A1338152, and they have likely reached an irreversible arrest state in which BCL-xL is the primary safeguard against apoptosis. This suggests that, although TIS deepening correlates with increased BCL-xL dependence, the requirement for mitochondrial priming varies as even minimally primed TIS cells (including *BAX*-deficient TIS cancer cells) can still be eliminated by BCL-xL-targeted senolytics.

**Figure 2:**
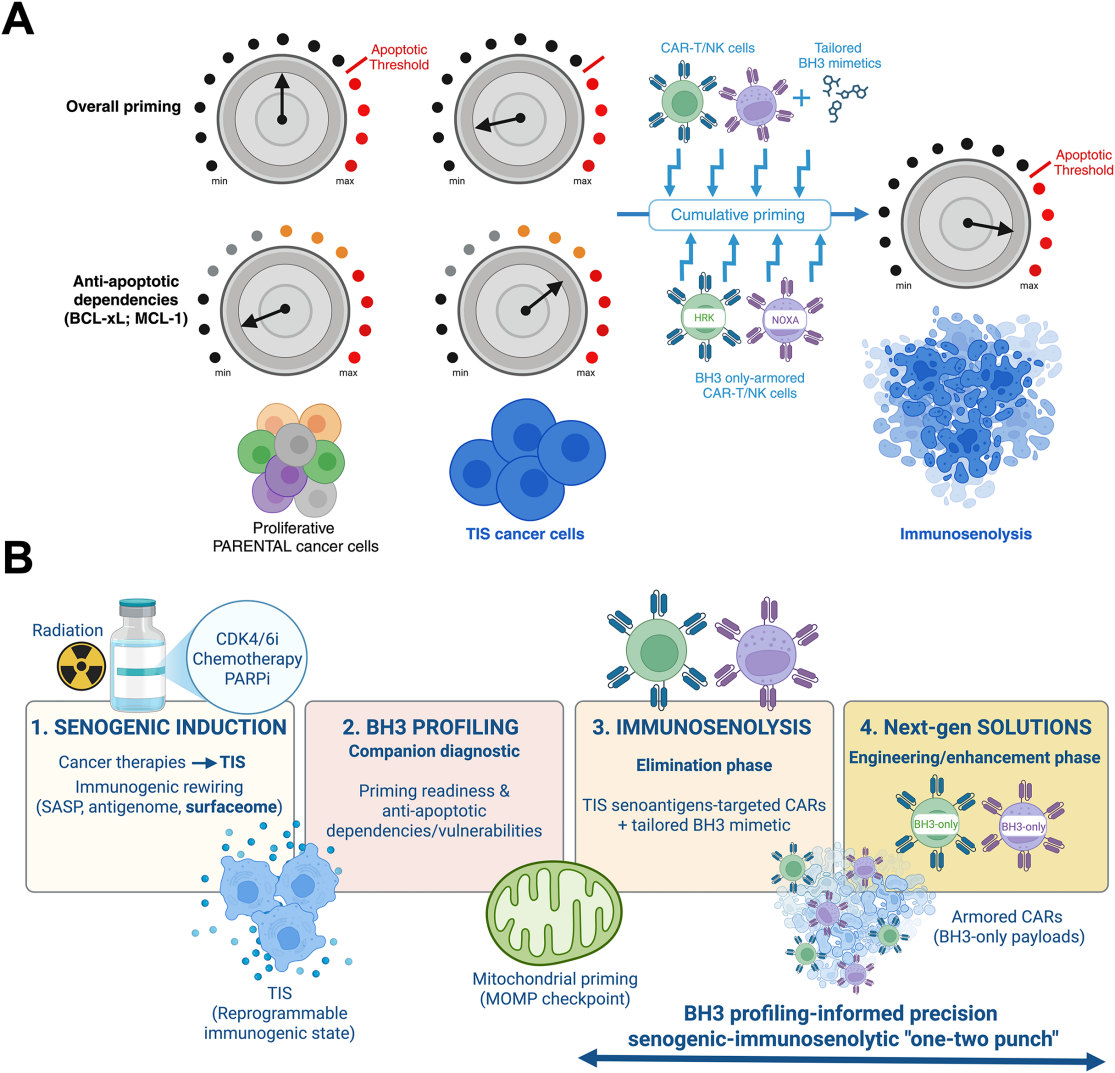
**(A)** The unique features of mitochondrial apoptotic priming in TIS cancer cells: An opportunity for guiding CAR-T/NK-driven immunosenolysis. TIS cancer cells exhibit a rewired mitochondrial apoptotic circuitry. In this circuitry, the permeabilization of the mitochondrial outer membrane (MOMP) acts as the decisive gateway (threshold) for immune-mediated cytolysis. At the low effector-to-target ratios typical of adoptive CAR-T/NK cell infusions, a single immune synapse is often insufficient to fully induce apoptosis in proliferating cancer cells. Instead, each transient engagement incrementally adjusts the mitochondrial rheostat of TIS cancer cells toward the apoptotic threshold. Thus, total exposure to successive immune contacts, rather than the intensity of a single encounter, dictates the likelihood of target elimination. This scenario is expected to be more refractory in TIS cancer cells, which are globally less primed for apoptosis than their proliferating precursors and depend on specific anti-apoptotic proteins (e.g., BCL-xL, MCL-1) for survival. Pharmacological BH3 mimetics that target specific anti-apoptotic BCL-2 family proteins can work together with the cumulative, priming synapse effect to selectively lower the mitochondrial threshold for apoptosis in TIS cells. This is expected to accelerate mitochondrial commitment to death in response to CAR-T/NK immunotherapy. Functional BH3 profiling can: **(i)** identify TIS-specific anti-apoptotic dependencies (e.g., BCL-xL versus MCL-1) and predict which BH3 mimetic will most effectively synergize with immune effector activity for a given TIS state, and **(ii)** guide the development of “armored” CAR-T/NK cells that carry synapse-restricted BH3-only modules to neutralize specific anti-apoptotic dependencies at the effector-target interface. This strategy ensures maximal alignment between pharmacological sensitization and immune effector activity. This framework positions mitochondria as both predictive sentinels and therapeutic checkpoints. It also underscores the need to integrate functional priming diagnostics with senogenic–immunosenolytic strategies. These strategies can transform the partial or abortive responses of TIS cancer cells to CAR-T/NK immunotherapy into durable, irreversible immunosenolysis. **(B)** Precision “one-two punch” designs integrating pharmacological and immunologic senolysis: A framework. This figure outlines a four-stage framework that integrates TIS, BH3 profiling, and engineered immunosenolysis in order to selectively eliminate cancer cells in next-generation “one-two punch” senogenic-immunosenolytic regimens. (1) Senogenic induction. Cancer therapies, including conventional genotoxic chemotherapy, CDK4/6 inhibitors, PARP inhibition, and ionizing radiation, drive tumor cells into TIS, a reprogrammable immunogenic state. TIS is characterized by secretory rewiring (SASP), altered antigen presentation (antigenome), and remodeling of the surfaceome (senoantigenome). (2) BH3 profiling. Functional companion diagnostics leverage BH3 profiling at defined treatment windows (pre- and post-TIS) to assess mitochondrial apoptotic priming and map anti-apoptotic dependencies and vulnerabilities within TIS cells. This process stratifies tumors for therapeutic responsiveness. (3) Immunosenolysis. During the elimination phase, TIS-associated antigens can be targeted by CARs. These CARs can be co-primed pharmacologically with tailored BH3 mimetics (or, ideally, degraders that spare platelets) to overcome apoptotic resistance and enforce MOMP in target TIS cancer cells. Since many TIS cancer cells depend on BCL-xL for survival, selective BCL-xL antagonism (or degrader strategies) are expected to work well with CAR-T/NK products. Dynamic adaptation, such as emergent MCL-1 rescue in response to BCL-xL-targeting strategies, can be countered with localized MCL-1 inhibition. (4) Next-generation solutions. Future engineering strategies include “armored” CAR-T/NK immunosenolytics that can deliver synapse-restricted BH3 only-modules (HRK/BAD to target BCL-xL or NOXA to target MCL-1) to neutralize BCL-xL/MCL-1 exactly where killing occurs, or TRUCK/SEAKER payloads that remodel the TIS-conditioned microenvironment. These strategies are expected to amplify apoptotic commitment and overcome residual resistance of the TIS reservoir. Multilayered safety logic can be incorporated to minimize off-tumor inhibition of anti-apoptotic BCL-2 proteins, including AND-gated antigen recognition, SASP-inducible promoters, and degron-tuned BH3 payloads. Senescence-associated immune evasion via PD-L1/PD-L2 could be addressed by incorporating dominant-negative or switch PD-1 receptors to maintain effector synapse fidelity and durability. This pragmatic trial schema could include correlative endpoints, such as single-cell BH3 maps of tumor and stroma senescence, CAR pharmacodynamics, imaging of mitochondrial-cytochrome C release imaging, clearance of TIS biomarkers, and monitoring of memory CARs persistence.

The new BH3 profiling-informed model of mitochondrial priming and anti-apoptotic dependencies in TIS cancer cells (**Figure 2A**) offers a mechanistic rationale for prioritizing BCL-xL-selective senolytics for clinical applications after TIS induction, particularly for tumors with transient senescence responses (e.g., palbociclib and olaparib TIS). In these cases, BCL-xL-selective senolytics [e.g., A1331852 and next-generation PROTAC BCL-xL degraders (108–112)] consistently outperform ABT-263/navitoclax. If TIS phenomena are expected to exhibit time- and cancer cell type-dependent heterogeneity in terms of deepening senescence, then administering senolytics at different time points after TIS induction could certainly influence the efficacy of one-two punch strategies in cancer treatment. Furthermore, the senescence phenotype is influenced more by cell type than by the senescence inducer (23). The apoptotic priming state of non-senescent parental cells foreshadows the senolytic susceptibility of TIS derivatives. This reveals an “inherited” mitochondrial memory that predicts responsiveness to senolytics (71). Thus, a comprehensive, tissue-based (or even cell-level) mapping of mitochondrial priming in the human body could serve as a proxy for the responsiveness of TIS cancer cells to senolytics. Testicular germ cell tumors (TGCTs) resemble human embryonic cells in that they have a hypersensitive apoptotic response to DNA damage due, at least in part, to increased mitochondrial priming (117, 118). Similarly, if mitochondrial apoptotic priming in cancers is linked to priming of the cell-of-origin, this correlation could be inherited by TIS cancer cell derivatives, providing senolytic sensitivity information from the beginning.

## 6 One-two punch senogenic-immunosenolytic regimens: from concept to clinical reality

Despite the conceptual appeal and mechanistic plausibility of using TIS to exploit the potency and selectivity of immunosenolytic cancer cell clearance, robust clinical proof of the “synthetic lethality 2.0” paradigm remains incipient. This is primarily due to three reasons: the reversibility and extent of the TIS phenotype in cancer tissues; the lack of easy-to-implement companion diagnostics; and the limitations of incorporating CAR-T/NK cell technology into solid tumor treatment.

### 6.1 TIS in cancer tissues: how long and how many?

Senescence is classically defined as the permanent cessation of cell division. However, entry into a TIS phenotype induced by radiation, chemotherapy, or targeted therapy is not necessarily a permanent endpoint, but rather a continuum toward full arrest of varying strength and depth. This continuum depends on senescence-associated maintenance mechanisms that, if not continuously stimulated, can allow cell cycle re-entry and override the TIS state, resulting in a proliferative post-TIS state that is not necessarily identical to the original state (119–124). However, it is important to note that targeting the acquired vulnerabilities of the transient senescent state or the determinants of senescence reversibility is sufficient to temporarily make the TIS phenotype susceptible to synthetic lethal approaches that can be exploited for therapeutic purposes to enforce the death of TIS cancer cells (116, 125).

TIS cancer cells have been identified in residual drug-resistant tumors, as well as in specimens with a partial or incomplete pathological response to neoadjuvant therapy (126–129). In breast cancer, 41% of tumors contained cells with active SA-β-gal after a course neoadjuvant therapy, which is significantly higher than the ∼2% frequency of SA-β-gal-positive cells in patients who did not undergo chemotherapy (121). Using a composite TIS signature involving p21^WAF1/Cip1+^, H3K9me3^+^, and lamin B1 loss, ∼41% of breast cancer samples with an incomplete or partial response to neoadjuvant chemotherapy showed marker expression consistent with a senescence-like phenotype (129). Detecting TIS based on a three-marker signature involving the downregulation of lamin B1 and Ki-67, as well as the upregulation of p16^INK4a^ revealed that 31% of breast cancer samples exhibited a shift toward a TIS-positive phenotype after exposure to neoadjuvant chemotherapy (130). Lamin B1 positivity decreased from 88% in pretreated malignant breast epithelium to 55% after exposure to neoadjuvant chemotherapy, indicating TIS induction (131). A pathology-level combination of four senescence markers (i.e., high lipofuscin and low Ki67 and p16^INK4a^/p21^WAF1/Cip1^ positivity) was present in ∼28% of non-small cell lung cancer samples treated with neoadjuvant therapy and in ∼32% of samples without neoadjuvant therapy (132). Among those tumors with a positive senescence signature, neoadjuvant therapy was associated with higher p16^INK4a^ levels (∼77% vs ∼53%) and higher lipofuscin, indicating a more pronounced senescent program post-therapy (132). In prostate cancer tissues, SA-β-gal expression increased with neoadjuvant therapy, primarily among intermediate-grade cancers with a more favorable clinical profile, and this phenotype become enriched over time (127). Androgen deprivation therapy, a widely used treatment for advanced prostate cancer, has also been shown to induce cellular senescence in prostate tumors (133).

Despite the fact that the reported percentages depend heavily on which markers are used (SA-β-gal versus p16/p21/lamin B1 versus lipofuscin panels) and on sample handling, the clinical signal that therapy induces a significant proportion of TIS cancer cells in residual disease is consistent across studies (131). Preclinical evidence strongly suggests that the efficacy of one-two punch regimens hinges on robust induction of TIS, which typically encompasses a substantial fraction of residual tumor cells, followed by efficient (>50-70%) senolytic clearance of this pro-survival compartment. Administering BH3 senolytics sequentially after senescence-inducing therapies markedly extends tumor suppression, linking durable therapeutic benefit to extensive senescence induction and nearly complete elimination of SASP-competent residual TIS cancer cells (14, 57). While most of the aforementioned clinical studies merely report a significant enrichment of senescent cells in post-therapy residual disease compared with untreated counterparts, assessable thresholds delineating the number of TIS cancer cells required for a beneficial (immuno)senolytic response remain undefined. A major limitation of the field is the absence of standardized, quantitative thresholds to define TIS burden in post-treatment tumor samples. This complicates cross-study comparisons and clinical translation. Emerging digital pathology and artificial intelligence-based morphometric inferences, and spatially resolved multi-omics would enable objective, spatially contextualized mapping of the TIS phenomenon at the single-cell level, overcoming the subjectivity inherent in conventional SA-β-gal or single-marker scoring. Integrating phenotypic, molecular, and spatial senescence signatures could establish a computational pathology framework that rigorously quantifies TIS dynamics and immunologic correlates across tumor contexts. This framework could ultimately inform the rational design, timing, and biomarker-driven optimization of senolytic interventions, particularly in the neoadjuvant setting, which is one of the most appropriate frameworks for evaluating the therapeutic impact of senotherapeutic-(immuno)senolytic approaches.

### 6.2 BH3 profiling as a companion diagnostic to guide CAR-T/NK immunosenolytics

We are accumulating evidence establishing BH3 profiling as a companion diagnostic that can be used to: (i) select the optimal BH3 mimetic, (ii) determine the dosing sequence (pretreatment versus cotreatment) to prevent T-cell toxicity, and (iii) predict and target anti-apoptotic dependencies to improve the depth and duration of CAR-T responses in hematologic malignancies. Early studies demonstrated high concordance between BH3-profiled apoptotic dependencies and the BH3 mimetic that best synergizes with the intrinsic-apoptosis killing capacity of CAR-T cells (134). This enables personalized selection of a BH3 mimetic/CAR-T combination that maximizes the therapeutic index for patients with T-cell lymphoma (135). In B-cell tumors, pretreatment with venetoclax/ABT-199 increases CD19 and pro-apoptotic protein levels, enhancing CD19 CAR-T cytotoxicity and persistence. However, concurrent or post-dosing harmed T cells. This underscores the importance of timing in BH3 profiling to inform BCL-2 reliance (136). In CD37^+^ T-cell lymphomas, BH3 profiling classified models as either MCL-1-dependent or BCL2/BCL-xL-dependent. Matching autologous CD37-directed CAR T cells (CAR-37) with AZD5991 (MCL1) or navitoclax/ABT-263 increased specific lysis and prolonged survival without impairing the CAR-T phenotype. This supports a profiling-guided combination paradigm (137). Engineering CAR-T cells to overexpress BCL-xL or venetoclax/ABT-199-resistant BCL2(G101V) improved expansion and persistence and maintained efficacy even with BH3 mimetics. This widens the therapeutic window for profiling-driven combinations (138). Across AML lines and primary blasts, cytotoxic T lymphocyte (CTL) killing converged on the mitochondrial pathway. BH3 mimetics lowered the apoptotic threshold and augmented T-cell cytotoxicity. MCL1 inhibition rescued venetoclax/ABT-199-resistant genotypes, once again arguing for dependency-guided selection of BH3 mimetics (139). Similarly, systematically characterizing mitochondrial priming, as measured by BH3 profiling assays, would enable prediction, monitoring, and improvement of the responses of TIS enriched-solid tumors to TIS-targeting CAR-T/NK cells.

Implementing BH3 profiling in solid tumors requires adapting this functional assay, which is traditionally optimized for hematological malignancies, to address the intrinsic heterogeneity, stromal content, and limited viable cell recovery of solid tumors. Dissociation protocols optimized to combine collagenase digestion with mechanical trituration may allow for the isolation of intact, mitochondria-bearing cells from small core or fine-needle aspirate biopsies in solid tumors (140). Integrating microfluidic and high-throughput dynamic BH3 profiling platforms (μDBP, HT-DBP) may enable the sensitive quantification of apoptotic priming from fewer than 10^4^ cells per condition (98, 141). Furthermore, developing fixable, flow-cytometric iBH3 assays based on cytochrome c retention in formaldehyde-fixed, permeabilized cells might enable the inclusion of stromal and immune compartments and allow for multiplexed phenotyping, cryopreservation, and compatibility with clinical cytometry pipelines (142, 143). Integrating BH3 profiling into patient-derived organoids or short-term explants would preserve native microenvironmental cues, thereby enhancing the physiological relevance and predictive accuracy for therapeutic responses (144). Coupling BH3 profiling with genomic annotation can help stratify tumors and guide BH3 mimetic–based combinations by identifying BH3 functional dependencies (e.g., BCL-XL addiction in RB1-deficient tumors (145)). The standardization of peptide panels, normalization controls, and automated data analysis pipelines has improved the reproducibility and throughput of BH3 profiling techniques, paving the way for CLIA-grade implementation.

These innovations could transform BH3 profiling from an experimental assay into a feasible functional precision oncology platform that quantifies apoptotic readiness and guides therapy selection in solid malignancies (91, 146–148). Because TIS cancer cells appear to respond to small-compound senolytics based on the mitochondrial apoptotic priming state of the pre-senescent parental cells, pretreatment tumor BH3 profiling could similarly serve as a predictive biomarker for immunosenolytic therapy response (70–73). However, we acknowledge that the clinical development of BH3 profiling and/or BH3 mimetic toolkits as companion diagnostics to optimize CAR-T/NK immunosenolytics as part of “one-two punch” treatments must be coupled with new technologies that can detect and isolate TIS cancer cells from mixed populations, including non-senescent normal cells and proliferative cancer cells, after treatment without destroying them. Recent advances have enabled the isolation of viable senescent cells from *in vivo* tissues through combined biochemical and genetic approaches (101). One strategy exploits the accumulation of lipofuscin, a complex of undegradable proteins, lipids, and metal aggregates formed by translational deregulation in response to stress, which is a hallmark of cellular senescence present in all types of senescent cells. Thus, tissues can be gently dissociated, and viable cells can be labeled with micelle-formulated GLF16. GLF16, a hydrophilic, live-cell-compatible derivative of Sudan Black B, targets lipofuscin and enables the detection and isolation of live senescent cells by flow cytometry directly from *in vivo* models (149, 150). High SA-β-gal activity can also be probed by using SPiDER-βGal in tissues. These tissues can be sorted by selecting the top signal tail (SPiDER^+^) to enrich senescent subsets (151). Multiparameter gating that integrates lipofuscin or SA-β-gal signals with cell size/granularity (FSC/SSC) can exclude dead/damaged cells and optionally incorporate p16/p21 expression or senescence-associated surface molecules, such as PD-L1. This further refines the specificity of isolation, in accordance with the 2024 consensus recommendations (152, 153). Other attempts to detect and isolate TIS cells using cell surface markers (DcR2, DPP4, and oxidized vimentin), fluorescent ubiquitin-based cell cycle indicator (FUCCI) technology, controlled release of the NIR fluorescent dye Nile Blue (NB), or density gradient-based separation of senescent cells are also promising (154–161), but are still far from being ready for *in vivo* validation.

### 6.3 Immunosenolytic use of the CAR-T/NK cell technology in solid tumors

*Bona fide* one-two punch senogenic-(immuno)senolytic regimens have yet to be validated in humans. Most of the early trials combining chemotherapy or targeted therapy with BH3 mimetics (e.g., ABT-263/navitoclax) occurred concurrently and were not designed to test the paradigm of inducing senescence followed by clearance of senescent cells. Though substantial evidence supports the “*make them senescent, then clear with senolytics*” strategy, it remains mostly remains in the preclinical stage (28). In triple-negative breast cancer (TNBC) human xenograft and orthotopic mouse models, combining the senogenic agent palbociclib with senolysis driven by ABT-263/navitoclax or a β-galactosidase-activated prodrug (“nav-Gal”) that mitigates navitoclax toxicity (i.e., thrombocytopenia/weight loss) delayed tumor growth and reduced metastases (162, 163). The Exactis-03 trial is the only ongoing clinical study explicitly testing the sequential senogenic-senolytic therapeutic logic (164).

Building on the finding that sequentially combining the PARPi olaparib with the senolytic ABT-263/navitoclax effectively controls tumors in ovarian and breast cancer models (116), the Exactis-03 trial (NCT05358639) is a multicenter, Phase I study that aims to implement a true one-two punch paradigm in clinical practice by administering a senogenic DNA-damage/PARP inhibitor (olaparib) followed by a senolytic BH3 mimetic (ABT-263/navitoclax). In this protocol, olaparib is administered as a monotherapy for two weeks to patients with *BRCA1/2* or *PALB2*-mutant TNBC or recurrent high-grade serous ovarian cancer. Biopsies are obtained at baseline and on days seven through twelve to assess senescence biomarkers and apoptosis biomarkers before introducing ABT-263/navitoclax intermittently in 28-day cycles. The primary objective of the trial is to define the recommended phase II dose (RP2D) of the olaparib/navitoclax combination, while monitoring for dose-limiting toxicities under a staggered schedule, which is intended to mitigate the occurrence of overlapping toxicities (164). Exploratory endpoints include a correlative evaluation of the SASP, modulation of pro- and anti-apoptotic BCL-2 family proteins, *ex vivo* organoid/multidrug tolerance (MDT) functional assays, and pharmacokinetics/biomarkers in serial blood samples.

Translating CAR-T and CAR-NK therapies from the fluid, antigen-uniform landscape of hematologic malignancies to the complex, immunosuppressive, and spatially fortified architecture of solid tumors presents significant biological and engineering challenges (**Box 1**). Not surprisingly, although the upregulation of actionable surface markers in TIS cancer cells established the basis for utilizing CAR-T or CAR-NK cells as live immunosenolytics (30–32), their application in an immunosenolytic sequence following customized senogenesis has not yet been examined in human cancer clinical trials. The only evidence supporting this approach comes from preclinical model. uPAR-targeted immunosenolytic CAR-T cells have been shown to efficiently clear senescent cells and improve fibrosis, metabolic dysfunction, cancer relapse and extend survival (30, 63, 75). These cells take advantage of the fact that uPAR is widely induced in epithelial, stromal, and stellate compartments across diverse senescence contexts. One example is TIS generated using a combination of the MEK inhibitor trametinib and the CDK4/6 inhibitor palbociclib in a *KRAS*-driven lung adenocarcinoma mouse model (30). Similarly, NKG2D-CAR-T cells recognize NKG2D ligands, which are upregulated by senescent cells and are naturally surveilled by CD8+ T cells and NK cells (32, 34, 165). The potency and durability with which uPAR- and NKG2D-CAR-T cells immunosenolytically clear senescent cells and improve metabolic and functional outcomes in mouse aging models provide a strong basis for future immunosenolytic second-punch trials. However, to date, no phase I/II trials targeting TIS cancer cells with CAR-T/NK cells have been registered or published.

Box 1 **CAR-T versus CAR-NK cells as immunosenolytics in solid tumors.**

Despite their transformative effectiveness in treating hematologic malignancies, CAR-T therapies have largely been ineffective against solid tumors due to multifactorial barriers. These barriers include poor intratumoral trafficking, antigen heterogeneity and escape, limited T-cell persistence, and profound immunosuppression within the TME (166–172). Despite potent preclinical models, many CAR-T trials in solid tumors have failed or stalled, crippling clinical efficacy. However, some successes have been seen in treating glioblastoma, neuroblastoma, and HER2-positive sarcomas. These successes demonstrate that precise antigen selection and “armored” CAR designs—those that secrete cytokines or incorporate checkpoint resistance (79, 173, 174)—can induce measurable tumor regression in select patients. CAR-NK cells have several advantages, including innate tumor recognition, a favorable cytokine profile, and reduced cytokine release syndrome (CRS) and immune effector cell-associated neurotoxicity syndrome (ICANS). They are also feasible of off-the-shelf allogeneic use. However, their clinical impact on solid tumors remains modest due to limited *in vivo* persistence, inefficient tumor infiltration, and suppression within the TME. This often results in only disease stabilization or partial responses in early solid-tumor studies (77, 175–179). The differential “success gap” between T and NK cells lies in the maturation and memory infrastructure of T cells versus the inherently shorter lifespan of NK cells. Nevertheless, engineering strategies, such as IL-15 armoring, chemokine receptors, and NK-specific signaling CAR scaffolds, show promise in bridging these functional divides and achieving durable antitumor responses in solid malignancies (180).

*In vivo* “reprogramming” of CAR-T/NK cells, which uses targeted gene delivery systems, such as viral vectors or lipid nanoparticles, to directly induce CAR expression in a patient’s endogenous T/NK cells. This method could improve persistence, trafficking, and tumor microenvironment adaptation. It could also circumvent issues with *ex vivo* manufacturing and exhaustion that limit efficacy in solid tumors (181–185). The systemic administration of cell-type–tropic lentiviral vectors in animal models has demonstrated the ability to reprogram endogenous lymphocytes *in vivo* to generate CAR-T/NK cells. Targeted nanoparticle nanoparticles platforms that enable the nonviral delivery of genetic material encoding CAR constructs or transcription factors. Compared to viral vectors, these platforms offer advantages such as transient expression, lower immunogenicity, and scalable manufacturing compared to viral vectors (186–188). These platforms should be adapted for *in vivo* T- and NK-cell transfection to demonstrate the feasibility of *in situ* T- and NK-cell engineering in solid tumors for clinical translation. Nevertheless, induced pluripotent stem cells (iPSCs)-CAR-NK technology enables the clonal generation of homogeneous, gene-edited NK cell populations from a renewable stem cell source. This technology overcomes donor variability and the manufacturing bottlenecks of primary cells. This type of platform supports precise genome engineering, such as site-specific CAR integration and the insertion of cytokine support modules. It allows for the efficient, large-scale production of functional, ready-to-use CAR-NK cells for rapid cell based-immunotherapy deployment (189–193).

Before choosing CAR-T versus CAR-NK cells as cell-based immunosenolytic therapies, several considerations must be addressed, including mechanistic fit, translational issues (e.g., manufacturing, safety, and pharmacokinetics/pharmacodynamics), and context-dependent liabilities.

**Antigen space and targeting logic**. If a cancer tissue exhibits a predominant, homogeneous TIS-associated marker following senescence-inducing treatment, a CAR-T or CAR-NK approach with a TIS senoantigen (e.g., uPAR)-specific single-chain variable fragment (scFv) domain should enable a single-antigen targeting strategy with high expected efficacy. However, if antigenicity is heterogeneous and stress-driven, employing NKG2D-CAR-like approaches in either T- or NK-cell backbones could mitigate the potential for target loss while exploiting senescence stress signals (194).

**Effector wiring and killing programs**. CAR-T cells exhibit potent T-cell receptor (TCR)-like cytotoxicity and cytokine production. They excel at detecting target cells and forming long-lived memory. While this is ideal for intermittent TIS cancer cell reseeding, it could lead to over-clearance if the targeted senoantigen temporarily serves a reparative role in normal tissues. CAR-NK cells combine CAR-driven activation of innate cytotoxicity (perforin/granzymes and TRAIL/FasL) with missing self-recognition. This provides multi-axis killing against antigen-low or antigen-heterogeneous TIS cancer cells. The same biology provides a built-in safety brake when inhibitory KIRs (killer Ig-like receptors) engage self-MHC. For instance, CAR-NK cells can integrate CAR signaling with endogenous NKG2D and DNAM-1 (CD226) signaling. NKG2D-CAR-T cells exploit this axis as well, but they lack NK inhibitory checkpoints and can sometimes amplify cytokine output (195, 196).

**Safety envelope**. The two most common and potentially life-threatening toxicities of CAR T-cell therapy are CRS and ICANS. Allogeneic, or “off-the-shelf”, CAR-T therapies have the potential to lower costs and expand access to a broader patient population. These therapies offer scalability, reduced production time, and enhanced, product consistency, and the elimination of the need for individualized manufacturing. However, their widespread use is limited by the risk of graft-versus-host disease (GvHD) (197). In immunosenolysis, where targets can be found on non-senescent reparative cells, high-potency CAR-T cells may be too powerful. CAR-NK cells, which do not express the TCR, are associated with a drastically lower CRS/ICANS and negligible GvHD. This enables safer outpatient regimens and dose fractionation, making them suitable for preventive or chronic immunosenolysis treatment (198). The sole caveat is a weaker *in vivo* expansion, which could render single-dose interventions ineffective.

**Pharmacokinetics and persistence**. Although long-lasting CAR-T cells are suitable for treating smoldering senescence, they may also eliminate beneficial, transient senescent cells that express the targeted senescence-associated antigen. Therefore, safety valves (e.g., suicide switches or ON-switch CARs) are essential (199, 200). Short-lived CAR-NK cells (lasting days to weeks) can be “pulsed” alongside senogenic therapy to eliminate TIS cancer cells during the high-damage window. This approach aligns with the desired pharmacokinetics of an immunosenolytic burst. iPSC-derived CAR-NK cells could facilitate the production of consistent, standardized batches (189–193).

**Trafficking and stromal access**. TIS cancer cells can reside in fibrotic stroma involving hepatic stellate cells and CAFs. uPAR-CAR-T cells have been shown to efficiently ablate uPAR-positive senescent cells and reverse fibrosis in liver and lung models (30, 75). This implies that, with appropriate cytokine engineering, CAR-T cells can penetrate the stroma to clear TIS cancer cells. TGFβ-resistant CAR-NK cells may have a functional advantage over unmodified CAR-NK cells in TGFβ-rich niches. Similarly, engineering CXCR2, CXCR4, and CCR2 into either platform will likely be necessary in scarred cancer tissues (201–204).

**Manufacturing, scale, and cost of goods**. Although autologous manufacturing of CAR-T cells is well-established, it may be logistically challenging for immunosenolytic applications (205). Allogeneic CAR-T cells reduce time and cost but also reintroduce allo-toxicity risks and require gene editing (e.g., TRAC/β2M) (206, 207). CAR-NK cells have multiple off-the-shelf sources, such as cord blood, NK-92, peripheral blood, and especially iPSC platforms (189–193). These sources align naturally with repeated dosing and population-scale use (208). However, ensuring consistent viability from batch-to-batch and during cryostorage remains a practical challenge under active optimization.

**Design specifics and safety considerations**. *Co-stimulatory domain choice.* CAR-T cells commonly use CD28/4-1BB to promote expansion and memory. CAR-NK cells benefit from NK-tuned endodomains (e.g., 2B4, DAP10/12, or 4-1BB chimeras), which align with the immunoreceptor tyrosine-based activation motif (ITAM), a key signal-transduction module that mediates activation upon receptor engagement in NK cells, to prevent anergic states (209). *Innate “backup” killing.* CAR-NK cells retain non-CAR cytotoxicity against low-antigen or antigen-negative TIS cancer cells escaping immunosurveillance, which may be superior in mosaic senescence fields after senogenic therapy. *Circuit breakers.* Due to the potential reparative roles of transient senescent cells, as well as the appearance of senoantigens/stress ligands on activated, non-senescent cells, both platforms would include time-bounded, senescence-state gating. Examples include ligand-gated ON switches and AND-gates with reporters that control CAR expression. These methods should be simply enough to standardize manufacturing processes (69, 179, 209–211). The risk of collateral stromal injury is likely lower with CAR-NK cells due to their transient persistence and inhibitor-receptor brakes. Large SASP bursts could amplify CRS in robustly expanding CAR-T cells, but the muted cytokine profile of CAR-NK cells could reduce this amplification loop.

CAR-T cells provide depth and persistence, making them ideal for long-term immunosurveillance against chronic senescence (re)seeding. This approach has already been validated preclinically in uPAR and NKG2D programs. If repeatable, clinically simple pulses are needed as a “scalpel” for peri-therapy immunosenolysis, CAR-NK cells may be the first pragmatic option for targeted immunosenolysis. They have a safer systemic profile and pose a lower risk of severe toxicities, such as CRS, neurotoxicity, and GvH. CAR-NK cells also have innate co-recognition of stresses senescent cells and a true off-the-self nature. This is coupled with the scalability of induced pluripotent stem cell technology. Thus, CAR-NK cells might surpass the challenges associated with autologous CAR-T manufacturing.

## 7 Conclusions and outlook

We propose a precision framework in which BH3 profiling can be performed before TIS and dynamically after senescence to stratify tumors based on their inherited mitochondrial priming and BCL-2 family dependencies and rescue plasticity. This framework informs both the senogenic primer and the immunosenolytic second hit (**Figures 2A, B**). For cancer lineages predicted to retain BCL-xL addiction after TIS, a senogenic pulse (e.g., a CDK4/6 inhibitor, a PARP inhibitor, or ionizing radiation) can be administered. Then, an infusion of CAR-T/NK cells targeting a specific TIS senomarker can be given during the period of maximum BCL-xL dependence. Co-priming with BCL-xL-specific antagonists (e.g., A-1331852 or a platelet-sparing BCL-xL degrader) can reduce the MOMP threshold selectively in target TIS cancer cells. If BH3 profiling indicates low global priming and strong HRK-sensitive depolarization, “armored” CAR-T/NK cells carrying modular BH3-only payloads can be used to neutralize BCL-xL at the immune synapse. Examples include granzyme B-shuttled HRK/BAD microdomains or split synNotch-gated BH3 translocators restricted to senomarker engagement (212, 213). This approach reproduces the pharmacological senolytic effect locally while sparing bystander tissues. For cohorts with emergent MCL-1 compensation in dynamic BH3 profiling, the cellular product can be upgraded to co-deliver NOXA (or granzyme B-NOXA fusions) or it can be combined with transient, tumor-confined MCL-1 antagonists while maintaining BCL-xL targeting. This eliminates escape probability without exhausting effectors.

T/NK-mediated killing is additive over serial, sub-lethal contacts and is limited by mitochondrial gating. Therefore, cell-delivered BH3 module and/or tailored pharmacological pro-apoptotics are expected to serve as synapse multipliers, converting partial hits into irreversible MOMP (**Figure 2A**). One way to strengthen the response is to remodel the TIS-conditioned microenvironment by integrating TRUCK logic (inducible IL-12/IL-18) to enhance antigen presentation and interferonized SASP (214, 215), while also avoiding exhaustion. Alternatively, SEAKER payloads that activate local prodrugs whose metabolites can either elevate priming or selectively degrade BCL-xL within TIS cancer niches can be developed (81, 216, 217). Since PD-L1/PD-L2 upregulation is a conserved TIS immune barrier, incorporating PD-1 dominant negative or switch receptors into the same construct could maintain synapse quality during senolysis (218–220). Using safe-gated AND-logic between TIS senomarkers, SASP-inducible promoters, and degron-tuned BH3 payloads could minimize off-tumor BCL-xL antagonism and mitigate thrombocytopenia associated with systemic BCL-xL blockade. Since pre-activated NK cells are intrinsically resistant to BH3 mimetics (87), NK-based products can be preferentially paired with systemic BCL-xL co-priming. This reserves CAR-T plus intracellular BH3 cargo for settings requiring maximal spatial specificity. BH3 profiling-informed “one-two punch” regimens combining tailored pharmacological priming with modular, armored CAR designs and TRUCK/SEAKER augmentation should enforce the apoptotic commitment of TIS reservoirs and deliver deeper, relapse-resistant remissions.

The complexity of the TIS-rewired mitochondrial circuitry explains why TIS cancer cells are refractory to most common cytotoxics but respond in a highly variably manner to (immuno)senolytic. The presented “one-two punch” paradigm involves first converts cancer cells into an immunogenic, primed state and then eradicates them through BH3 profiling-informed immunosenolysis. This approach provides a logical plan for the next generation of precision immunosenolytic oncology, which could transform partial or sub-lethal immune responses into irreversible senolysis (**Figure 2B**). This precision framework could therapeutically translate the senogenic “first punch” from a relapse-prone byproduct (123, 124) into a predictive and therapeutically exploitable checkpoint that would enable durable, relapse-resistant “second punch” immunosenolytic responses to adoptive CAR-T/NK immunotherapy.

## Data Availability

The original contributions presented in the study are included in the article/supplementary material. Further inquiries can be directed to the corresponding authors.

## Glossary

ADCs: Antibody-drug conjugates
BAD: Bcl-2-associated death
BAX: Bcl-2-associated X protein
BCL-2: B-cell leukemia/lymphoma 2
BH3: Bcl-2 homology 3 domain
BID: BH3-interacting domain death agonist
BIM: B-cell lymphoma 2-like protein 11
BiTES: Bi-specific T-cell engager
BMF: Bcl-2 modifyingfFactor
CAR-NK: chimeric antigen receptor (CAR)-engineered NK cells
CAR-T: chimeric antigen receptor (CAR)-engineered T cells
CAR: chimeric antigen receptor (CAR)-engineered T and NK cells
CDK4/6: Cyclin-dependent kinase 4 and 6
cGAS/STING: Cyclic GMP-AMP synthase/stimulator of interferon genes
CLIA: Clinical laboratory improvement amendments
CRS: Cytokine release syndrome
CSF1: Colony stimulating factor 1
DNAM-1: DNAX accessory molecule-1
FADD: Fas-associated death domain protein
FDA: U.S. Food and Drug Administration
HRK: Harakiri
ICANS: Immune effector cell-associated neurotoxicity syndrome
Immune IFN: Interferon
MCL-1: Myeloid cell leukemia 1
ITAM: immunoreceptor tyrosine-based activation motif
MHC: Major histocompatibility complex
MOMP: Mitochondrial outer membrane permeabilization
NKG2D: Natural killer group 2D
NOXA: latin for “damage”
NSG: NOD-scid IL2rγnull
PARP: poly (ADP-ribose) polymerase
PD-1: Programmed cell death protein-1
PD-L1: Programmed death-ligand 1
PD-L2: Programmed death-ligand 2
PROTAC: Proteolysis TArgeting Chimera
PUMA: p53 upregulated modulator of apoptosis
SASP: Senescence-associated secretory phenotype
SCCNVs: Senescent cancer cell-derived nanovesicle
SEAKERs: Synthetic enzyme-armed Killer cells
STAT: Signal transducer and activator of transcription
TIS: Therapy-induced senescence
TNF: Tumor necrosis factor
TRUCKs: T cells redirected for universal cytokine-mediated killing
uPAR: Urokinase-type plasminogen activator receptor.
